# Nano‐liposomal encapsulation of *Artemisia aucheri* phenolics as a potential phytobiotic against *Campylobacter jejuni* infection in mice

**DOI:** 10.1002/fsn3.2921

**Published:** 2022-05-17

**Authors:** Asmae Mehdizadeh, Ehsan Karimi, Ehsan Oskoueian

**Affiliations:** ^1^ Department of Biology Mashhad Branch Islamic Azad University Mashhad Iran; ^2^ Department of Research and Development Arka Industrial Cluster Mashhad Iran

**Keywords:** antibiotic alternatives, drug delivery, nanoliposome, natural products, phytogenic

## Abstract

**Background:**

*Artemisia aucheri* contains antibacterial phenolic compounds. The current work was implemented to evaluate the effectiveness of a nanoliposome‐encapsulated phenolic‐rich fraction (PRF‐NLs), as a dietary phytobiotic derived from *Artemisia aucheri's* areal parts, on the inhibition of enteropathogenic *Campylobacter jejuni* (*C. jejuni*) infection in mice.

**Methods:**

The phenolic‐rich fraction was loaded into the nanoliposome structure to obtain a nanometer‐scale size liposome with homogenous dispersion. Next, 40 white male balb/c mice were assigned to 4 treatment groups. The PRF‐NLs antibacterial potential was evaluated by evaluating the blood parameters, liver lipid peroxidation, and gene expression profiling in the mice challenged by *C. jejuni* infection.

**Results:**

Mice infected by *C. jejuni* showed impairment in food intake, weight gain, liver function, ileum morphometric features, and ileum tissue inflammation. The diet of fortified food with the nonencapsulated and nanoliposome‐encapsulated phenolic compounds was found to improve these parameters at 10 mg TPC/kg BW/day concentration. Our data indicated that the nanoliposome‐encapsulated PRF was more effective in promoting the health parameters in mice as compared to nonencapsulated PRF.

**Conclusion:**

It could be concluded that the liposomal encapsulation can promote the solubility, availability, and effectiveness of *Artemisia aucheri* phenolic compounds playing a key role as phytobiotic in mice intervened by enteropathogenic *C. jejuni*.

## Graphical abstract

There is an increasing interest in medicinal plants as natural alternatives to synthetic drugs to combat antibiotic‐resistant microorganisms. Liposomal formulation products are extensively utilized in a wide spectrum of life science technologies, i.e., healthcare, biosensors and biomedicines, cosmetics, veterinary medicine, and agricultural and pharmaceutical industries. Nano‐liposomal encapsulation technique is a feasible approach to secure plant bioactive compounds such as phenolic compounds to increase chemical/biological stability thereof. The liposomal encapsulation phenolics from *A. aucheri* aerial parts investigated in the present study could be a promising and sustainable phytobiotic approach to combat *Campylobacter jejuni* infections without any side effects.

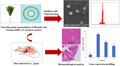

## 1 INTRODUCTION

The efficiency of a drug depends on its structure and delivery mechanism in carrying the drug into the body, permeating the barriers, and ultimately reaching the predetermined area (Rahman et al., 2013). There are several pharmaceutical transportation systems, used in medical and biological research, to facilitate drug absorption and greater uptake in cells. Drug transport across a biological barrier is dependent on its solubility (Sur et al., 2019). Research has shown that nearly two‐fifths of the available drugs in the market have poor water solubility, an issue that limits sufficient absorption, which leads to low therapeutic efficacy (Chaudhari & Akamanchi, 2019; Chen et al., 2019).

The liposome, as one of the best choices among the available transportation systems, has a spherical‐shaped vesicle comprised of one or more phospholipid bilayers. The ability to encapsulate hydrophilic or hydrophobic drugs makes liposomes good candidates for drug delivery (Lee & Thompson, 2017; Olusanya et al., 2018). Nanotechnology has great attention nowadays and opens up a wide array of application in different filed including agriculture and medicine (Pramanik et al., 2020). Nanoliposomes are submicron bilayer lipid vesicles that encapsulate bioactive agents and increase drug performance by improving their solubility, stability, and bioavailability. The ability to target specific cells allows nanoliposomes to avoid unwanted interactions with nonspecific molecules (Bulbake et al., 2017; Wang et al., 2018). Nanoliposomes have recently been used to design and construct delivery systems for the natural bioactive compounds, namely phenolic compounds. Phenolic compounds have undeniable biological impacts on human health (Esfanjani et al., 2018; Rafiee et al., 2017). They cast a major role in the suppression mechanisms (e.g., carcinogen inactivation, apoptosis induction, angiogenesis suppression, and antioxidation) of several types of cancer (Heleno et al., 2015). Moreover, the configuration and the quantity of the structural hydroxyl groups in their structure concern their antioxidant and antimicrobial capacity (Carocho & CFR Ferreira, 2013). Also, several research articles have documented the polyphenols bioactivities.

*Artemisia aucheri* (Asteraceae), a perennial aromatic medicinal plant, is widely distributed throughout Asia, Europe, and America. The plant is known for its broad spectrum of bioactivity and natural bioactive compounds, videlicet polyphenols, flavonoids, terpenoids, and phenylpropanoids (Hussain et al., 2017; Kazemi, 2015). Several studies have underpinned the significance of *A*. *aucheri's* biological properties and therapeutic effects on human health. Therefore, the current work aimed to (a) produce a liposome‐encapsulated *A*. *aucheri* polyphenol and (b) investigate its phytobiotic potential against *Campylobacter jejuni* (*C. jejuni*) infection in mice.

## 2 MATERIALS AND METHOD

### 2.1 Plant material and reagent

We purchased the *A*. *aucheri* fresh aerial parts from Mashhad herbal medicine market, Iran. The *C. jejuni* was purchased from the microbial culture collection of the Islamic Azad University of Mashhad, Iran. In this study, soybean lecithin was obtained from Sigma Aldrich. The RNA extraction and cDNA synthesis kits as well as SYBER Green master mix were all bought from Biofact company. The other material and reagents not mentioned here were from Daejung.

### 2.2 Fractionation

The areal parts of the *A*. *aucheri* plant were cleaned with sterile distilled water and dried in shadow for 2 weeks at room temperature. The dried plant material was finely ground (powder form) using a grinder mill. Then, 100 g of the dried powder was extracted with 900 ml of aqueous methanol (80% (v/v)) and 100 ml of 6 M HCl using the reflux method for 2 h (Karimi et al., 2019). Then, the extract was filtered, and the filtrate was concentrated and evaporated at the temperature of 60°C by using a rotary evaporator (Buchi). In the next step, the extract was fractionated using a separating funnel and different solvents including hexane, chloroform, ethyl acetate, n‐butanol, and water‐based as described earlier Oskoueian et al. (2020). Upon fractionation, the supernatant was filtered and concentrated using a vacuumed rotary evaporator. The total phenolic compound (TPC) evaluation of each fraction was carried out by adding 0.1 ml of the extract, 2.5 ml of Folin‐Ciocalteu reagent (1:10 v/v), and 2 ml of 7.5% sodium carbonate into a test tube covered with aluminum foil. The test tubes were vortexed and the absorbance was measured at 765 nm as described earlier by Oskoueian et al. (2020). The results were expressed as milligrams of gallic acid equivalents (GAE) per gram of dry weight. The fraction containing the highest phenolic content is named a phenolic‐rich fraction (PRF).

### 2.3 Preparation of nano‐liposomal carriers

To prepare the nanoliposomes, 4 g of lecithin was added to 196 g of 80°C hot water and stirred for 2 h. In the next step, the PRF was added and stirred for 2 additional hours to attain 2000 ppm as the final concentration. Ultimately, the solution was sonicated for 6 min; the acquired nanoliposome‐loaded PRF was synthesized for further characterization (Beyrami et al., 2020).

### 2.4 Characterization of nanoliposomes

After diluting the nanoliposomes‐loaded PRF in water (1:20), the dynamic light scattering (DLS) protocol was used to determine the zeta potential of the particles. Field Emission Scanning Electron Microscopy (FESEM) was additionally employed to determine the nanoliposomes’ size dimensions. Malvern Zetasizer Nano ZS was recruited to analyze the measurements three times.

### 2.5 Nanoliposomes phenolic profiling with HPLC

Reversed‐phase HPLC (RP‐HPLC) analysis was conducted in compliance with Karimi et al. (2019) and Oskoueian et al. (2011) to investigate the phenolic profiling of the nanoliposomes‐loaded PRF from *A*. *aucheri*. We used two solvents: solvent A (deionized water) and solvent B (acetonitrile). Before injection, we equilibrated the column with 85% of solvent A and 15% of solvent B for 15 min. After 50 min, the ratio of solvent B was increased to 85%. At minute 55, the ratio of solvent B was declined to 15%; this ratio was maintained for 1 h to run the subsequent analysis with a flow rate of 0.6 ml/min. As described previously, gallic acid, syringic acid, vanillic acid, salicylic acid, caffeic acid, pyrogallol, catechin, cinnamic acid, ellagic acid, naringin, chrysin, and ferulic acid were considered phenolic standards (Karimi et al., 2018).

### 2.6 In vivo experiment

Forty (20–25 g) white male Balb/c mice were purchased from the Razi Vaccine and Serum Research Institute of Mashhad, Iran. All mice were kept in individual cages at 23 ± 1°C and 58% ± 10% humidity; they were exposed to 12‐h light/dark periods for 7 days for adaptation to laboratory conditions. Ten mice were assigned to each group in 4 groups overall. The mice had access to tap water and were fed from the standard pellet diet produced by Javaneh Khorasan, Mashhad, Iran. The experimental treatments were:

Treatment 1: typical diet.

Treatment 2: typical diet + infection by *C. jejuni* on day 21.

Treatment 3: typical diet supplemented by nonencapsulated PRF (10 mg TPC/kg BW/day) + infection by *C. jejuni* on day 21.

T4: Normal diet supplemented by nanoliposome‐encapsulated PRF (10 mg TPC/kg BW/day) + infection by *C. jejuni* on day 21.

Animals received the treatments for 4 weeks. All samples were gavaged orally using 10^8^ cfu of *C. jejuni* on day 21. We monitored the mice on daily basis for general health and the quantity of diet. On day 28 (the end of the experiment), pentobarbital‐HCL (50 mg/kg, i.p.) was used to euthanize the mice. Immediately, the blood, liver, and ileum samples were excluded and liver enzyme analysis, lipid peroxidation assay, gene expression analysis, and the morphometric analysis of the ileum were evaluated. The mice were weighed on altered occasions throughout the experiment: the beginning, the middle, and the end of the process. All animal experiments were implemented following the research ethics approved by the Islamic Azad University of Mashhad.

### 2.7 Liver enzymes and lipid peroxidation assay

The liver enzymes, such as SGOT, SGPT, and ALP, were determined in the serum. The liver's lipid peroxidation was examined based on Shafaei et al. (2020). The samples’ absorbance was read at 532 nm; the output was presented as a percentage relative to the control.

### 2.8 Histopathology analysis

The mice were euthanized, and their livers, kidneys, and ileum were washed in the normal saline. Eventually, the separated organs were preserved in 10% buffered formalin (in 0.1 M sodium phosphate buffer, pH7) and paraffinized, sliced for staining following Shafaei et al. (2020) hematoxylin/eosin protocol.

### 2.9 Validation of gene expression

To evaluate the molecular mechanism of action of PRF nanoliposomes in promoting the health status of the mice, we determined the expressions of fundamental biomarker genes, namely COX2, iNOS, SOD, and GPx. Liquid nitrogen was used to freeze the ileum tissues immediately upon sampling. The frozen tissues were then crushed in mortar and pestle in the presence of liquid nitrogen to prepare for RNA extraction using an RNA extraction kit. Next, we synthesized the cDNA libraries entirely using a cDNA synthesis kit. Furthermore, the sets of primer sequences were used to investigate the expression level of key genes as well as the beta‐actin as the housekeeping gene. Noteworthy, we employed the SYBR Green PCR Master Mix in a comparative qPCR (Roche Diagnostics). The (target) genes were amplified accordingly at 95°C for 5 min (1X), 95°C for 20 s, 56°C for 25 s, and 72°C for 30 s (35×). The genes expressions were normalized to beta‐actin (as the reference gene) and the relevant genes in the control group (Kathirvel et al., 2010). See Table 1 for the primers’ characteristics.

**TABLE 1:** The primer applied in this research

Gene	Forward (5′→3′)	Reverse (5′→3′)	References
SOD	gagacctgggcaatgtgact	gtttactgcgcaatcccaat	Wang et al. (2010)
GPx	ctcatgaccgaccccaagta	cccaccaggaacttctcaaa	Yamamoto et al. (2016)
COX2	caagcagtggcaaaggcctcca	ggcacttgcattgatggtggct	Jain et al. (2008)
iNOS	caccttggagttcacccagt	accactcgtacttgggatgc	Gao et al. (2015)
β‐actin	cctgaaccctaaggccaacc	cagctgtggtggtgaagctg	Heger et al. (2016)

### 2.10 Analysis of ileum microbial population

The qPCR was performed to characterize the population of *C. jejuni* in the ileum. Briefly, to extract the DNA from 1 ml of the ileum digesta, we used the QIAamp DNA Stool extraction kit (QIAGEN). Table 2 illustrates the primers used in this study. In addition, we adopted 2^−∆∆Ct^ method to analyze the data from the qPCR to identify the fold changes in the *C. jejuni* population (Feng et al., 2010). The 2^−∆∆Ct^ displays the difference between the ΔCt values at the pre‐ and postbacterial challenge. The cycle threshold (also, Ct) is known to be the point where the fluorescence rises above the background. The qPCR system software was recruited to determine the Ct values according to the threshold line tuned manually above the noninformative fluorescent data.

**TABLE 2:** PCR primers list used for ileum microbial population

Bacteria	Forward (5′→3′)	Reverse (5′→3′)	References
*C. jejuni*	cgggatagttatagtattgaagtt	gaaggagcataataggatcttg	Razzuoli et al. (2018)
Total bacteria	cggcaacgagcgcaaccc	ccattgtagcacgtgtgtagcc	Denman and McSweeney (2006)

### 2.11 Procedures of data analysis

All experiments were performed in triplicates, and statistical analysis of data was expressed as Means ± standard deviations (*SD*). We ran Duncan's Multiple Range Test to determine the significance of the means differences (*p* < .05).

## 3 RESULTS AND DISCUSSION

### 3.1 Fractionation and identification of phenolic compounds

The TPC found within different fractions of *A*. *aucheri* indicated that the phenolic compounds varied from 4.7 ± 2.02 to 52.4 ± 1.45 mg gallic acid equivalent (GAE)/100 g dried fraction. Whereas the ethyl acetate fraction (52.4 ± 1.45 mg GAE/g DM) was found to contain the highest amount of phenolic content, the hexane fraction showed the lowest amount (4.7 ± 2.02 mg GAE/g DM). Several early studies indicated that the phenolic compounds are moderately polar compounds; that is why they tend to accumulate in the fraction of medium polarity such as ethyl acetate (Kaur et al., 2008; Olatunji et al., 2017). The ethyl acetate fraction, as a PRF, was selected for the encapsulation process and further evaluations. In line with our findings, previous studies (Abdelwahab et al., 2010; Kaur et al., 2008) reported that while various polarity solvents were used to extract phenolic compounds, the highest TPC was found in the ethyl acetate fraction.

### 3.2 Size, zeta potential, and morphology of PRF‐NLs

**FIGURE 1:**
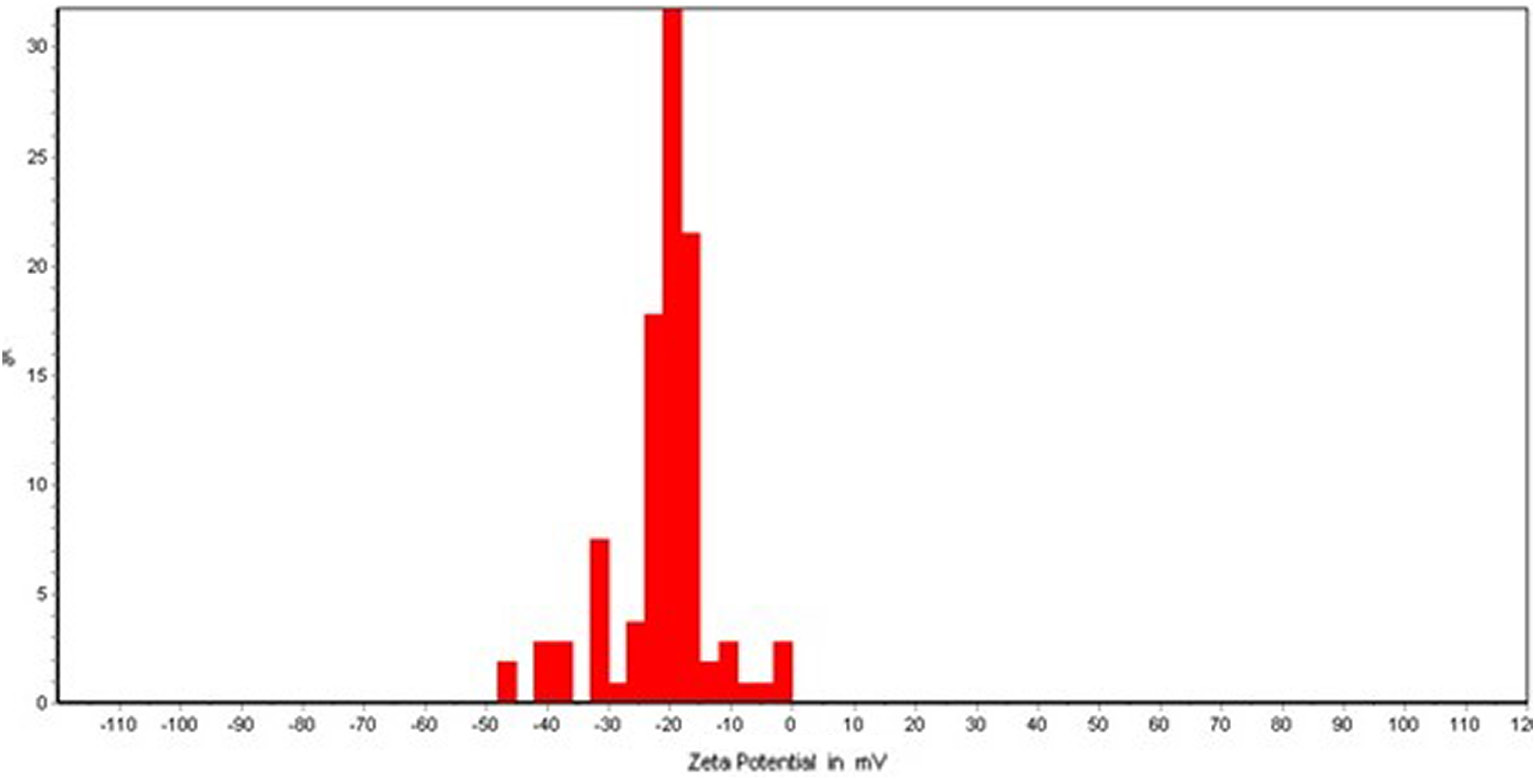
The zeta potential of liposomes containing Artemisia aucheri phenolics‐rich fraction

**FIGURE 2:**
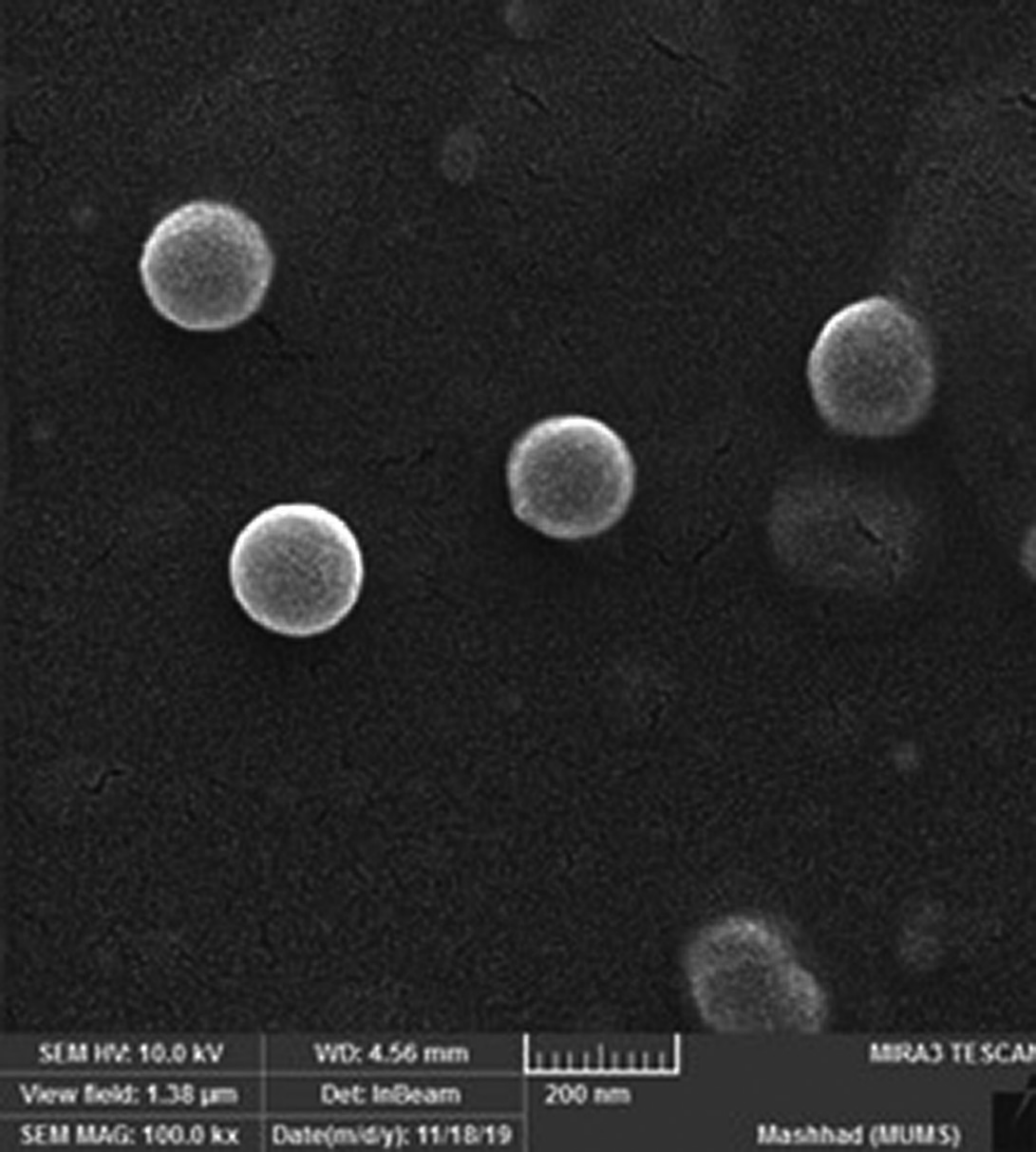
The SEM analysis of liposomes containing Artemisia aucheri phenolics‐rich fraction

Characteristics such as size, zeta potential, and morphology of the nanoliposomes are considered important parameters for stability, bioavailability, and the release behavior of nanoparticles. As demonstrated in Table 3, the PRF encapsulated in liposome carrier had a nanometer size (170.3 ± 6.89 nm); hence, it was named phenolic‐rich fraction‐loaded nanoliposome (PRF‐NLs). The PRF‐NLs exhibited a negative surface charge (−21.04 mV) (see Figure 1), indicating low to moderate stability based on Kumar and Dixit’s (2017) classification. Furthermore, the PDI value (0.258) showed that PRF‐NLs had high homogeneity and narrow particle size distribution. Figure 2 illustrates the PRF‐NLs morphology, demonstrating its spherical shape and distribution.

**TABLE 3:** DLS analysis and surface charge of *Artemisia aucheri* nanoliposome

Particle size (nm)	Polydispersity index	Zeta potential (mV)
170.3 ± 6.89	0.258	−21.04

### 3.3 TPC and HPLC analysis of PRF‐NLs

According to our data, the TPC of the PRF‐NLs was 18.7 ± 2.18 mg GAE/g DM. Phenolic compounds profiling by HPLC unveiled various types of natural phenolic compounds (see Table 4). Among others, caffeic acid (705 µg/g DW) and chrysin (983 µg/g DW) were the dominant polyphenols found in the PRF‐NLs of *A*. *aucheri*.

**TABLE 4:** Phenolic profiling of *Artemisia aucheri* nanoliposome

Phenolic compounds contents (µg/g DW)						
GA	CA	SA	CA	CT	EA	CH
185 ± 0.32	702.5 ± 1.3	208 ± 1.2	311 ± 3.5	226 ± 1.8	162 ± 4.1	983 ± 3.4

Note

The analysis was performed in triplicates.

Abbreviations: CA, cinnamic acid; CF, caffeic acid; CH, chrysin; CT, catechin; EA, ellagic acid; GA, gallic acid; SA, syringic acid.

### 3.4 Mice trial

According to Table 5, mice with *C. jejuni* infection (T2) demonstrated a considerable decrease (*p* < .05) in the average daily weight gain and dietary intake in comparison to their un‐infected counterparts (T1). Supplementation of the PRF to the infected (with *C. jejuni*) groups (T3 and T4), in the form of nonencapsulated and nanoliposome‐encapsulated, significantly improved (*p* < .05) these parameters. These results revealed that supplementing liposome‐encapsulated phenolic compounds was more effective in improving daily weight gain and food intake in the infected mice than those supplemented with nonencapsulated. Such difference could be ascribed to liposomal encapsulation, which enhanced the solubility, bioavailability, and the effectiveness of the antioxidant and antibacterial activity of the bioactive compounds in the PRF.

**TABLE 5:** The body weight changes and feed intake analysis

Average	T1	T2	T3	T4	*SEM*
Average daily gain (g)	0.19^a^	0.14^c^	0.16^bc^	0.18^ab^	0.06
Average daily feed intake (g)	3.1^a^	2.5^c^	2.7^bc^	2.9^b^	0.12

Note

Different letters in the same row indicate significant difference (*p* < .05). The analysis was performed in triplicates.

Abbreviations: T1, normal diet; T2, normal diet + infected by *C. jejuni* on day 21; T3, normal diet enriched by a nonencapsulated phenolic‐rich fraction (10 mg TPC/kg BW/day) + infected by *C. jejuni* on day 21; T4, normal diet enriched by a nanoliposome‐encapsulated phenolic‐rich fraction (10 mg TPC/kg BW/day) + infected by *C. jejuni* on day 21.

### 3.5 Liver enzyme and lipid peroxidation analysis

According to Table 6, mice treated with *C*. *jejuni* (T2) showed a remarkable increase (*p* < .05) in liver enzymes and lipid peroxidation as compared to the control group (T1), indicating the effect of infection on the liver malfunction. Moreover, these results unraveled that *C. jejuni* infection could trigger oxidative stress in the liver. The SGOT, SGPT, and ALP enzymes and lipid peroxidation were considerably modulated (*p* < .05) by dietary supplementation of nonencapsulated and nanoliposome‐encapsulated PRFs. These observations concorded to the results obtained from daily weight gain and food intake, underscoring the effectiveness of nanoliposome‐encapsulated PRF in the alleviation of liver enzymes and lipid peroxidation in comparison to the nonencapsulated PRF.

**TABLE 6:** Key enzymes and lipid peroxidation results in mice liver

Liver enzymes (IU/L)	T1	T2	T3	T4	*SEM*
SGOT	139.5^d^	219.8^a^	185.9^b^	163.5^c^	6.89
SGPT	98.8^d^	220.6^a^	159.7^b^	131.2^c^	7.49
ALP	154^d^	190.0^a^	164^b^	118.5^c^	6.29
MDA (%)*	100.0^d^	158.8^a^	139.6^b^	125.4^c^	6.73

Note

Different letters in the same row indicate significant difference (*p* < .05).

Abbreviations: T1, normal diet; T2, normal diet + infected by *C. jejuni* on day 21; T3, normal diet enriched by a nonencapsulated phenolic‐rich fraction (10 mg TPC/kg BW/day) + infected by *C. jejuni* on day 21; T4, normal diet enriched by a nanoliposome‐encapsulated phenolic‐rich fraction (10 mg TPC/kg BW/day) + infected by *C. jejuni* on day 21.

* Changes relative to control.

The alleviation of liver enzymes and lipid peroxidation by using phytobiotic through dietary regimen could be associated to the antioxidant and antibacterial activity of the gallic acid, caffeic acid, syringic acid, cinnamic acid, catechin, ellagic acid, and chrysin present in the developed phytobiotic. On the other hand, the liposomal encapsulation could improve the solubility and bioavailability of the phenolic compounds, making nanoliposome encapsulation more efficacious than their nonencapsulated counterpart. Consistent with our findings, previous research has pointed to the phenolic compounds’ hepatoprotective activity against enteropathogens endotoxins (Saha et al., 2019).

### 3.6 Histological and morphometric analysis

**FIGURE 3:**
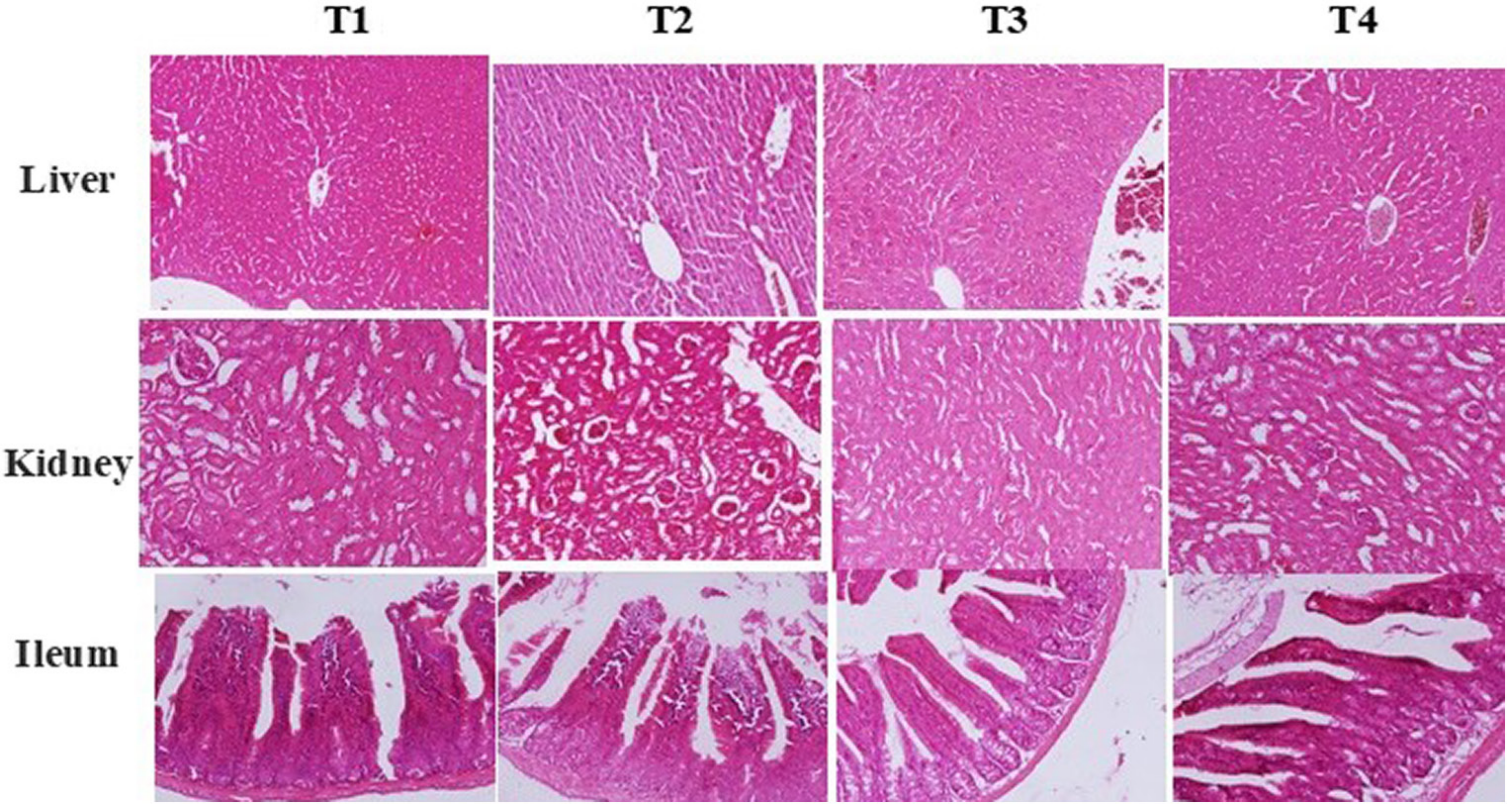
Histopathological analysis of liver, kidney, and ileum of the mice received different treatments. T1, normal diet; T2, normal diet + infected by *C. jejuni* on day 21; T3, normal diet enriched by a nonencapsulated phenolic rich fraction (10 mg TPC/kg BW/day) + infected by *C. jejuni* on day 21; T4, normal diet enriched by a nanoliposome‐encapsulated phenolic rich fraction (10 mg TPC/kg BW/day) + infected by *C. jejuni* on day 21

Figure 3 illustrates the histological architecture of the mice liver, kidney, and ileum tissues after different treatments. The results revealed that the *C. jejuni* infection and dietary treatment with nanoliposome‐encapsulated and nonencapsulated PRFs resulted in an insignificant change in the kidney and liver tissues. In addition, the morphometric analysis unveiled that mice infected by *C. jejuni* (T2) intervention experienced a reduction in the ileal villus height, villus width, and the quantity of goblet immune cells and a meaningful increase (*p* < .05) in the crypt depth.

The villus height, villus width, and the crypt depth could be considerably improved (*p* < .05) by the dietary supplementation of 10 mg TPC/kg BW/day of nonencapsulated and nanoliposome‐encapsulated PRFs. The improvement in the morphostructural characteristics of the intestine resulted in better absorption of nutrients and subsequently enhance the daily weight gain. These observations were consistent with the early study conducted by Jamroz et al. (2006) who reported the stimulatory effects of plant bioactive compounds on growth and development of villus, increase in the production of mucus on the inner wall of the intestine which prevents enteropathogens colonization.

The results observed in this study were in line with earlier studies highlighting the contribution of the plant's bioactive compounds in promoting the morphometric structure of the ileum under enteropathogens treated/untreated conditions in rabbits (Pogány Simonová et al., 2020), pigs (Nofrarias et al., 2006), rats (Erlwanger & Cooper, 2008), and broiler chickens (Khan et al., 2017) (Table 7).

**TABLE 7:** Morphometric characteristic of ileum

	T1	T2	T3	T4	*SEM*
Villus height (µm)	440.8^bc^	429.9^c^	449.6^b^	457.7^a^	4.68
Villus width (µm)	97.6^c^	86.2^d^	105.3^b^	117.7^a^	3.82
Crypt depth (µm)	150.5^ab^	156.7^a^	145.9^c^	137.4^d^	3.18
Mean number of goblet cells	5.1^a^	3.5^c^	4.3^b^	4.2^b^	0.48

Note

Different letters in the same row indicate significant difference (*p* < .05)

Abbreviations: T1, normal diet; T2, normal diet + infected by *C. jejuni* on day 21; T3, normal diet enriched by a nonencapsulated phenolic‐rich fraction (10 mg TPC/kg BW/day) + infected by *C. jejuni* on day 21; T4, normal diet enriched by a nanoliposome‐encapsulated phenolic‐rich fraction (10 mg TPC/kg BW/day) + infected by *C. jejuni* on day 21.

### 3.7 Gene expression analysis

Table 8 presents the expression level of the inflammatory and antioxidant genes within the ileum tissue. The *C. jejuni* (T2) treatment down‐regulated (*p* < .05) SOD and GPx (as major biomarkers of cellular redox potential) gene expression and up‐regulated (*p* < .05) COX2 and iNOS (as inflammatory genes) expression in comparison to the unchallenged group (T1). Supplementing 10 mg TPC/kg BW/day of nonencapsulated and nanoliposome‐encapsulated PRFs up‐regulated significantly (*p* < .05) the expression of SOD and GPx and down‐regulated (*p* < .05) COX2 and iNOS genes expression in a meaningful way. It could be understood that the regulation of the inflammation and antioxidant‐related genes could be related to the phenolic compounds’ anti‐antioxidant and anti‐inflammatory activities of gallic acid, caffeic acid, syringic acid, cinnamic acid, catechin, ellagic acid, and chrysin presence in the PRF (Ambriz‐Pérez et al., 2016; Rubió et al., 2013; Shahidi & Ambigaipalan, 2015). Hence, the regulation of inflammation‐ and antioxidant‐related genes in the current study might be due to the presence of bioactive phenolics compounds in the nonencapsulated and nanoliposome‐encapsulated PRF. Apart from that, the higher intestinal solubility, absorption of nanoliposome‐encapsulated PRF resulted in significant (*p* < .05) down‐regulation in the inflammation‐ and up‐regulation in antioxidant‐related genes as compared to the mice received nonencapsulated PRF.

**TABLE 8:** Gene expression pattern in the ileum tissue

Fold changes					*SEM*
Genes	T1	T2	T3	T4	
SOD	1.0^c^	−2.1^d^	+1.9^b^	+2.6^a^	0.11
GPx	1.0^c^	−1.9^d^	+0.9^b^	+1.8^a^	0.09
COX2	1.0^d^	+6.4^a^	+3.8^b^	+2.1^c^	0.07
iNOS	1.0^d^	+4.3^a^	+3.6^b^	+2.3^c^	0.12

Note

Different letters in the same column indicate significant difference (*p* < .05). The analysis was performed in triplicates

Abbreviations: T1, normal diet; T2, normal diet + infected by *C. jejuni* on day 21; T3, normal diet enriched by a nonencapsulated phenolic‐rich fraction (10 mg TPC/kg BW/day) + infected by *C. jejuni* on day 21; T4, normal diet enriched by a nanoliposome‐encapsulated phenolic‐rich fraction (10 mg TPC/kg BW/day) + infected by *C. jejuni* on day 21.

### 3.8 Population analysis of C. jejuni

**FIGURE 4:**
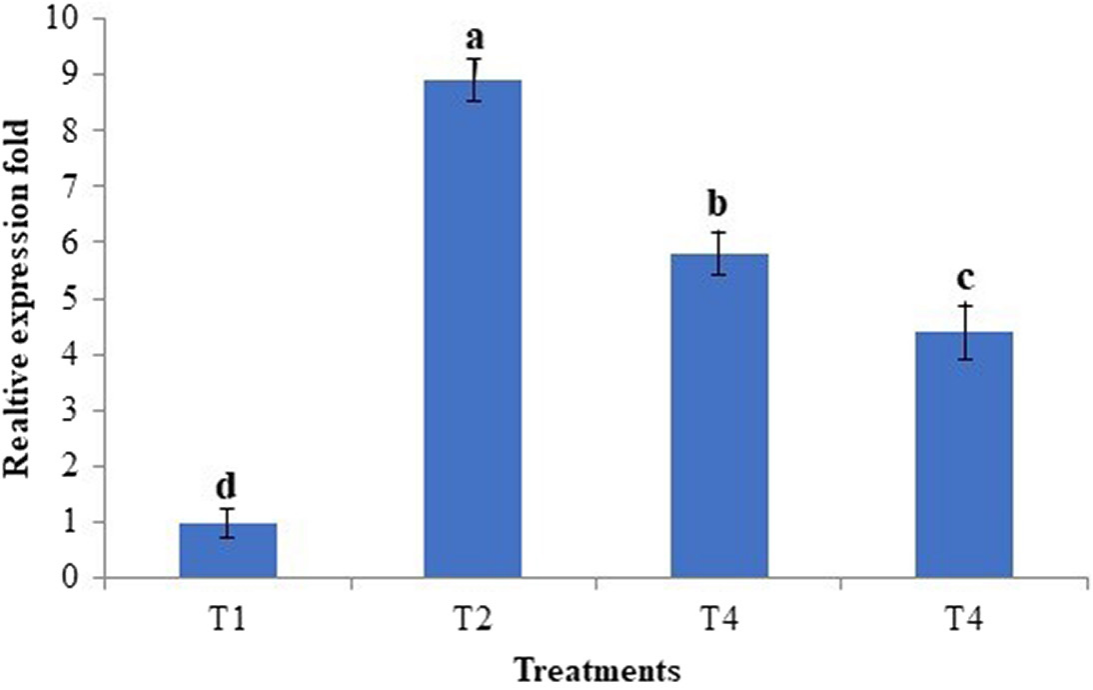
Relative quantification of *C. jejuni* population in ileum digesta. T1, normal diet; T2, normal diet +infected by *C. jejuni* on day 21; T3, normal diet enriched by a nonencapsulated phenolic rich fraction (10 mg TPC/kg BW/day) + infected by *C. jejuni* on day 21; T4, normal diet enriched by a nanoliposomeencapsulated phenolic rich fraction (10 mg TPC/kg BW/day) + infected by *C. jejuni* on day 21

Figure 4 indicates the relative alterations within the quantity of *C. jejuni* in the ileum digesta after receiving different treatments. The *C. jejuni* challenge significantly incremented the *C. jejuni* population (*p* < .05) by 8.9 folds in the ileum digesta compared to the unchallenged group. The inclusion of nonencapsulated PRF and nanoliposome‐encapsulated PRF considerably reduced (*p* < .05) the number of C. *jejuni* in the ileum digesta by 5.8 and 4.4 folds.

These results revealed that nanoliposome‐encapsulated PRF more effectively modulated the enteropathogenic *C. jejuni* in the ileum as compared to the nonencapsulated PRF. The higher antibacterial activity of nanoliposome‐encapsulated PRF as compared to the nonencapsulated PRF could be related to the higher intestinal solubility and absorption of nanoliposome‐encapsulated PRF. Thereby, the nanoliposome‐encapsulated PRF could be considered as a natural antibiotic alternative called phytobiotic to prevent intestinal infection caused by *C. jejuni*. Besides, these bioactive phenolics present in the PRF stimulate the production of intestinal mucus which then creates a thick layer of mucus on the inner wall of the ileum and reduces the possible colonization of *C. jejuni* and resulted in the reduced population of *C. jejuni* (Bouarab‐Chibane et al., 2019; Jamroz et al., 2006).

## 4 CONCLUSION

According to the obtained results, nonencapsulated and nanoliposome‐encapsulated PRFs of *A*. *aucheri*, with a concentration of 10 mg TPC/kg BW/day, could inhibit the pathogen population and improve the mice health parameters when challenged by enteropathogenic *C. jejuni*. The delivery of nanoliposome‐encapsulated phenolic complexes was found more active in improving the health parameters than the nonencapsulated phenolic compounds. Thus, it could be concluded that liposomal encapsulation may promote the solubility, availability, and effectiveness of *A*. *aucheri* phenolic compounds. As a phytobiotic, *A*. *aucheri* phenolics are found to cast a prominent role in the inhibition of enteropathogenic *C. jejuni* infection in mice. The isolation and encapsulation of phenolics individually and evaluation of their phytobiotic potential against *C. jejuni* are recommended for the future works.

## CONFLICT OF INTEREST

The authors have declared that there are no competing interests.

## ETHICAL APPROVAL

All protocols to conduct an in vivo study were described in compliance with ARRIVE guidelines. All animal investigations were carried out based on the ethical principles approved by the Islamic Azad University of Mashhad, Iran (code no: IR.IAU.MSHD.REC.1399.012).
